# Diabetes and pregnancy: national trends over a 15 year period

**DOI:** 10.1007/s00125-017-4529-3

**Published:** 2018-01-11

**Authors:** Sharon T. Mackin, Scott M. Nelson, Joannes J. Kerssens, Rachael Wood, Sarah Wild, Helen M. Colhoun, Graham P. Leese, Sam Philip, Robert S. Lindsay, on behalf of the SDRN Epidemiology Group

**Affiliations:** 1 Institute of Cardiovascular and Medical Sciences, British Heart Foundation Glasgow Cardiovascular Research Centre, University of Glasgow, 126 University Place, Glasgow, G12 8TA UK; 2 School of Medicine, University of Glasgow, Glasgow, UK; 3 Farr Institute Scotland, Nine Edinburgh Bioquarter, Edinburgh, UK; 4 ISD Scotland, Edinburgh, UK; 5 Institute of Genetics and Molecular Medicine, University of Edinburgh, Edinburgh, UK; 6 Department of Diabetes, Ninewells Hospital and Medical School, Dundee, UK; 7 Department of Diabetes, Aberdeen Royal Infirmary, Aberdeen, UK; 8 Glasgow Royal Infirmary, Glasgow, UK

**Keywords:** Diabetes, Epidemiology, Perinatal, Pregnancy, Trends, Type 1 diabetes, Type 2 diabetes

## Abstract

**Aims/hypothesis:**

We aimed to examine time trends in national perinatal outcomes in pregnancies complicated by pre-existing type 1 or type 2 diabetes.

**Methods:**

We analysed episode-level data on all obstetric inpatient delivery events (live or stillbirth) between 1 April 1998 and 31 March 2013 (*n* = 813,921) using the Scottish Morbidity Record (SMR02). Pregnancies to mothers with type 1 (*n* = 3229) and type 2 (*n* = 1452) diabetes were identified from the national diabetes database (Scottish Care Information-Diabetes), and perinatal outcomes were compared among women with type 1 diabetes, type 2 diabetes and those without diabetes.

**Results:**

The number of pregnancies complicated by diabetes increased significantly, by 44% in type 1 diabetes and 90% in type 2 diabetes, across the 15 years examined, to rates of 1 in 210 and 1 in 504 deliveries, respectively. Compared with women without diabetes, delivery occurred 2.6 weeks earlier (type 1 diabetes 36.7 ± 2.3 weeks) and 2 weeks earlier (type 2 diabetes 37.3 ± 2.4 weeks), respectively, showing significant reductions for both type 1 (from 36.7 weeks to 36.4 weeks, *p* = 0.03) and type 2 (from 38.0 weeks to 37.2 weeks, *p* < 0.001) diabetes across the time period. The proportions of preterm delivery were markedly increased in women with diabetes (35.3% type 1 diabetes, 21.8% type 2 diabetes, 6.1% without diabetes; *p* < 0.0001), and these proportions increased with time for both groups (*p* < 0.005). Proportions of elective Caesarean sections (29.4% type 1 diabetes, 30.5% type 2 diabetes, 9.6% without diabetes) and emergency Caesarean sections (38.3% type 1 diabetes, 29.1% type 2 diabetes, 14.6% without diabetes) were greatly increased in women with diabetes and increased over time except for stable rates of emergency Caesarean section in type 1 diabetes. Gestational age-, sex- and parity-adjusted *z* score for birthweight (1.33 ± 1.34; *p* < 0.001) were higher in type 1 diabetes and increased over time from 1.22 to 1.47 (*p* < 0.001). Birthweight was also increased in type 2 diabetes (0.94 ± 1.34; *p* < 0.001) but did not alter with time. There were 65 perinatal deaths in offspring of mothers with type 1 diabetes and 39 to mothers with type 2 diabetes, representing perinatal mortality rates of 20.1 (95% CI 14.7, 24.3) and 26.9 (16.7, 32.9) per 1000 births, respectively, and rates 3.1 and 4.2 times, respectively, those observed in the non-diabetic population (*p* < 0.001). Stillbirth rates in type 1 and type 2 diabetes were 4.0-fold and 5.1-fold that in the non-diabetic population (*p* < 0.001). Perinatal mortality and stillbirth rates showed no significant fall over time despite small falls in the rates for the non-diabetic population.

**Conclusions/interpretation:**

Women with diabetes are receiving increased intervention in pregnancy (earlier delivery, increased Caesarean section rates), but despite this, higher birthweights are being recorded. Improvements in rates of stillbirth seen in the general population are not being reflected in changes in stillbirth or perinatal mortality in our population with diabetes.

**Electronic supplementary material:**

The online version of this article (10.1007/s00125-017-4529-3) contains peer-reviewed but unedited supplementary material, which is available to authorised users.


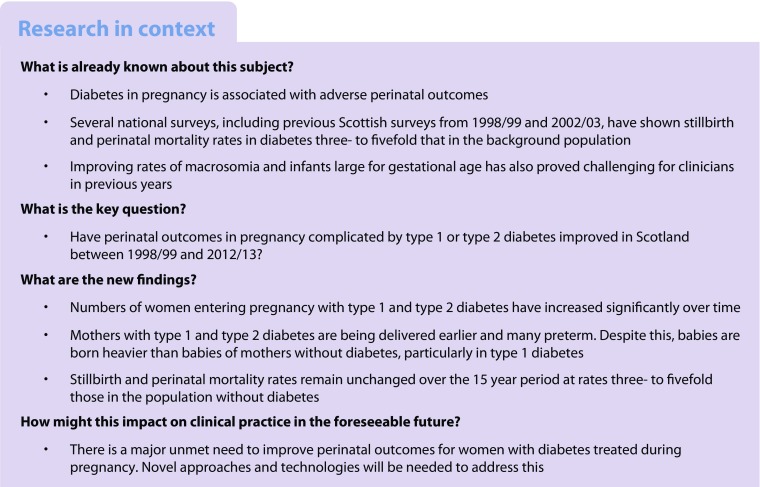


## Introduction

Type 1 and type 2 diabetes confer significant additional risks in pregnancy, with increased rates of stillbirth, perinatal mortality, macrosomia, prematurity and operative delivery [1–6]. Results of several national surveys between 1990 and 2008, including previous paper-based national surveys in Scotland in 1998/1999 and 2003/2004 [7, 8] showed that, despite marked improvement before these years [9], rates of stillbirth and perinatal mortality among women with diabetes prior to pregnancy continued to be broadly 3–5 times those of the non-diabetic population [1, 10–13]. Whether rates of stillbirth and perinatal mortality have changed thereafter has been less clear—although, importantly, the most recent data for England and Wales suggest a marked reduction in stillbirth rates for women with type 1 diabetes from 25.8 (18.3 to 33.3) per 1000 live births in 2002/2003 [2] to 10.7 in 2015 [14], and from 29.2 (16.3 to 42.2) to 10.7 for women with type 2 diabetes. Data for birthweight have shown less dramatic historical trends, with relatively stable birthweight up to 1990 for type 1 diabetes [9].

In this study, we analysed nationally collected data from Scotland to examine whether perinatal outcomes for mothers with type 1 and type 2 diabetes were changing.

## Methods

### Study population

We linked the Scottish Morbidity Record 02 (SMR02) to the Scottish Care Information-Diabetes (SCI-Diabetes) database. SMR02 includes information on all women discharged from Scottish maternity hospitals, including maternal and infant demographics, clinical management and obstetric complications. It includes a standard measure of social deprivation (Scottish Index of Multiple Deprivation [SIMD]) [15]. It is subject to regular quality assurance, and the most recent validation of a 4.4% sample (*n* = 2531) with case records showed that all the data items used in our study were more than 90% complete and accurate [16]. Gestational age has been confirmed by ultrasound in early pregnancy in more than 95% of women in the UK since the early 1990s [17]. SCI-Diabetes collates demographic and clinical data for people with a diagnosis of diabetes in Scotland. From 2004 onwards, population coverage of the register has been 99.5% [18].

Diabetes type was defined in the clinical record of SCI-Diabetes. This was further refined by algorithm if contradicted by available information on age at diagnosis and prescription history, reassigning as type 2 diabetes if there had been more than 1 year of receiving no diabetes medication or solely non-metformin oral glucose-lowering agents, and reassigning as type 2 diabetes if no contradictory prescription history was present and the individual had been diagnosed at less than 30 years of age [19, 20]. Diagnosis of diabetes has previously been validated in SCI-Diabetes against inpatient records, with greater than 99% accuracy [18]. Gestational diabetes was not always recorded and is not considered here.

Approval for records access and linkage was obtained from the Caldicott guardians of all Health Boards in Scotland, the Privacy Advisory Committee of the Information Services Division (ISD) of NHS National Services Scotland and the national multicentre research ethics committee. In keeping with the ethics application, no individual consent was taken but identifiable information was not supplied to researchers. Information was stored and analysed in a pseudonymised format.

### Inclusion and exclusion criteria

We obtained SMR02 data for all infants delivered between 1 April 1998 and 31 March 2013. Analyses were restricted to births in girls or women older than 10 years who delivered between 24 and 44 weeks of gestation inclusive.

### Definitions

The *z* score for birthweight was calculated in cells based on gestational age, sex and parity using a set of standard LMS-tables derived from all Scottish births from 1998 to 2003 [21].

Stillbirth was defined as a child born after 24 weeks’ gestation who did not breathe or show signs of life, while perinatal mortality was defined as the combination of this and death in the first week of life.

Infants were defined as ‘large for gestational age’ (LGA) if they were born weighing above the 90th centile corrected for gestational age, sex and parity.

### Statistical analyses

Pregnancy outcomes in women by diabetes status were compared using ANOVA or logistic regression with post hoc testing between groups (ANOVA), or by *χ*^2^ test as appropriate. Data are expressed as means ± SD unless stated otherwise. Trends for changes in outcome over time were assessed by ANOVA with terms for type of diabetes, time and interaction between these tested. Binomial distribution was used to obtain 95% CI for ORs in trend analysis. All analyses were performed using SPSS version 22.0 (IBM Corp., Armonk, NY, USA).

Data are presented for all deliveries for maternal variables, stillbirth and perinatal mortality (*n* = 813,634), but confined to singletons for mode of delivery, gestational age at delivery including preterm birth and birthweight variables (*n* = 801,271).

## Results

A total of 813,921 deliveries were recorded in Scotland across the audit period, of which 38 were excluded from the study owing to the unknown vital status of the infant. Among these, 4681 (0.6%) were to mothers with pregestational diabetes, 3229 (69%) of whom had had type 1 diabetes for an average 13.2 years and 1452 (31%) type 2 diabetes for an average of 3.3 years (Table 1). A further 249 mothers were linked to SCI-Diabetes with another diagnosis (including gestational diabetes, impaired glucose tolerance or maturity-onset diabetes of the young) and were not considered further.

**Table 1:** Maternal characteristics and obstetric outcomes over the 1998–2013 period

Variable	Type 1 diabetes	Type 2 diabetes	No pregestational diabetes^a^
Number of ongoing pregnancies after 24 weeks	3229	1452	808,953
Maternal age at delivery, years^a^	29.2 ± 5.7**^, †††^	32.8 ± 5.5***	28.8 ± 6.0
Duration of diabetes, years^a^	13.2 ± 8.4^†††^	3.3 ± 3.6	
Parity^a^			
Nulliparous, % (*n*)	50.4 (1604)***^, †††^	30.5 (438)***	45.9 (368,476)
Multiparous, % (*n*)	49.6 (1585)	69.5 (998)	54.1 (434,672)
SIMD, % (*n*)^a^			
SIMD1 most deprived	25.0 (807)^‡, §§§^	31.0 (449)^‡‡‡^	25.8 (208,221)
SIMD2	21.1 (680)	22.2 (321)	20.7 (167,327)
SIMD3	20.3 (656)	20.0 (290)	18.6 (149,684)
SIMD4	17.9 (576)	13.7 (198)	17.7 (143,065)
SIMD5	15.7 (507)	13.1 (190)	17.2 (138,477)
Maternal smoking in pregnancy, % (*n*)^b^	21.5 (635)	21.3 (281)	23.5 (174,749)
Stillbirths, *n* (*n* per 1000 births)	63 (19.5) ***	36 (24.8)***	3966 (4.9)
Perinatal mortality, *n* (*n* per 1000 births)	65 (20.1) ***	39 (26.9) ***	5154 (6.4)
Multiple pregnancy, % (*n*)	1.2 (40)	1.4 (21)	1.5 (12,166)
Singleton babies, % (*n*)	98.8 (3189)	98.6 (1431)	98.5 (796,649)
Mode of delivery ^b^			
ELCS, % (*n*)	29.4 (956)***	30.5 (445)***	9.6 (76,776)
EMCS, % (*n*)	38.3 (1251)***^, †††^	29.1 (430)***	14.6 (118,284)
Mean birthweight, g	3466.7 ± 802.8^†††^	3474.4 ± 793.1***	3398.8 ± 587.9
LGA, % (*n*)^b^	50.9 (1623)***^, †††^	38.4 (549) ***	10.5 (84,141)
*z* score for birthweight^b^	1.33 ± 1.34***^, †††^	0.94 ± 1.34***	0.04 ± 1.01
Gestation at delivery, weeks^b^	36.7 ± 2.3***^, †††^	37.3 ± 2.4***	39.3 ± 2.0
Preterm delivery (<37 weeks), % (*n*)^b^	35.3 (1126) ***^, †††^	21.8 (311)***	6.1 (48,576)
Very preterm delivery (<32 weeks), % (*n*)^b^	3.8 (121)***	3.2 (46) ***	1.1 (8760)

Values are presented as mean ± SD or *n* (95% CI) unless indicated otherwise

^a^Excluding 38 pregnancies missing vital status and 249 with other diabetes

^b^Missing data: Maternal age missing in ten cases, duration of diabetes missing in 56 cases, parity missing in 5861 cases, SIMD missing in 2186 cases, maternal smoking missing in 65,866 cases, mode of delivery missing in 23 cases, birthweight missing in six cases, gestational age at delivery missing in 412 cases

**p < 0.01, ***p < 0.001 vs no diabetes; ^†††^*p* < 0.001 vs type 2 diabetes (Pearson’s *χ*^2^ test for categorical variables, ANOVA for continuous variables)

^‡^*p* < 0.05, ^‡‡‡^*p* < 0.001 vs no diabetes across classification (Pearson’s *χ*^2^ test)

^§§§^*p* < 0.001 vs type 2 diabetes across classification (Pearson’s *χ*^2^ test)

**Fig. 1:**
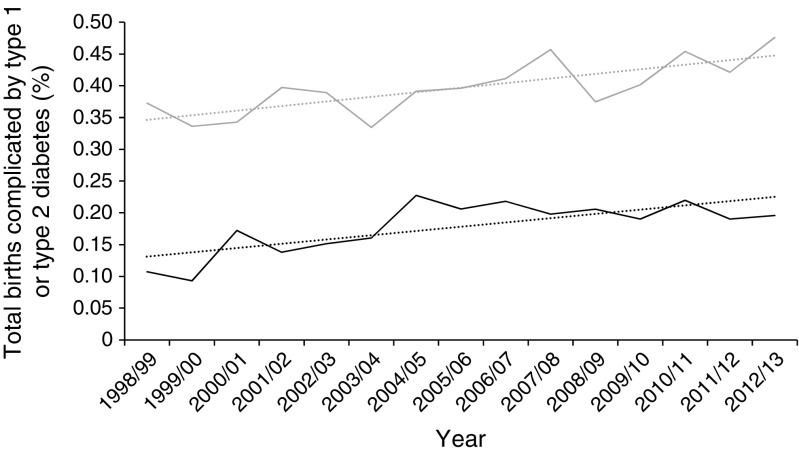
Trends in absolute numbers of pregnancies complicated by diabetes. Light grey, type 1 diabetes; black, type 2 diabetes; dotted lines in corresponding colours show trends

There were 104 perinatal deaths in the offspring of mothers with diabetes across the 15 years (65 for type 1 diabetes, 39 for type 2 diabetes), representing rates rates 3.1 and 4.2 times those observed in the non-diabetic population (*p* < 0.001; Table 1). Stillbirth rates in type 1 and type 2 diabetes were 4.0-fold and 5.1-fold that in the non-diabetic population (*p* < 0.001), and occurred at a mean gestational age of 33.6 and 34.1 weeks, respectively. Perinatal mortality rates were unchanged over time (see electronic supplementary material [ESM] Fig. 1).

The number of births to mothers with type 1 diabetes increased significantly from 1998/1999 to 2012/2013 (from 205 to 264 deliveries), with a larger increase in type 2 diabetes (from 59 to 110) (Fig. 1). Both increases were significant (*p* < 0.001) and suggested a 44% increase in deliveries to mothers with type 1 diabetes and a 90% increase in type 2 diabetes in fitted models.

Mothers with type 1 diabetes were on average 0.4 years older (29.2 ± 5.7 years) and more likely to be nulliparous than the general population (Table 1). By contrast, mothers with type 2 diabetes were 4 years older than mothers without diabetes, had higher rates of deprivation and were more likely to have had a previous pregnancy (Table 1).

There were marked differences in pregnancy outcomes in women with diabetes compared with non-diabetic women. On average, women with type 1 diabetes and type 2 diabetes were delivered 2.6 weeks and 2 weeks earlier than women without diabetes (Table 1). The proportions of preterm (less than 37 weeks’ gestation) and very preterm (less than 32 weeks’ gestation) delivery were increased in women with diabetes, with a fivefold increase in preterm delivery in type 1 diabetes (Table 1). The proportions of elective Caesarean section (ELCS) and emergency Caesarean section (EMCS) were greatly increased, with 67.7% of women with type 1 diabetes and 59.6% of women with type 2 diabetes undergoing operative delivery (Table 1). Despite significantly earlier delivery, mean absolute birthweights in type 1 and type 2 diabetes were higher than in those without diabetes (68.7 g heavier in type 1 diabetes, 76 g heavier in type 2 diabetes) but only formally statistically significant in type 2 diabetes (*p <* 0.001) (Table 1). When adjusted for gestational age, parity and sex, these differences were dramatic, with the offspring of mothers with type 1 diabetes born at average weights 1.33 SD above those of the non-diabetic population, and offspring of mothers with type 2 diabetes averaging 0.94 SD above the non-diabetic population (Table 1). Around 51% of babies born to mothers with type 1 diabetes and 38% born to mothers with type 2 diabetes were defined as LGA (Table 1).

One of the strengths of our data is the ability to look at trends over time. Across the 15 years, mean maternal age at delivery increased by 0.6 years in mothers with type 1 diabetes and 1.6 years in mothers with type 2 diabetes (from 32.4 to 34 years on average) (ESM Fig. 2). Maternal age increased by 0.9 years in mothers without diabetes, and while the trend to increasing age was highly significant (*p* < 0.001), it was no different in mothers with diabetes (*p* = 0.56). Mean duration of diabetes also increased, from 12.4 to 14.6 years in type 1 diabetes and from 2.6 to 3.8 years in type 2 diabetes (*p* < 0.001).

**Fig. 2:**
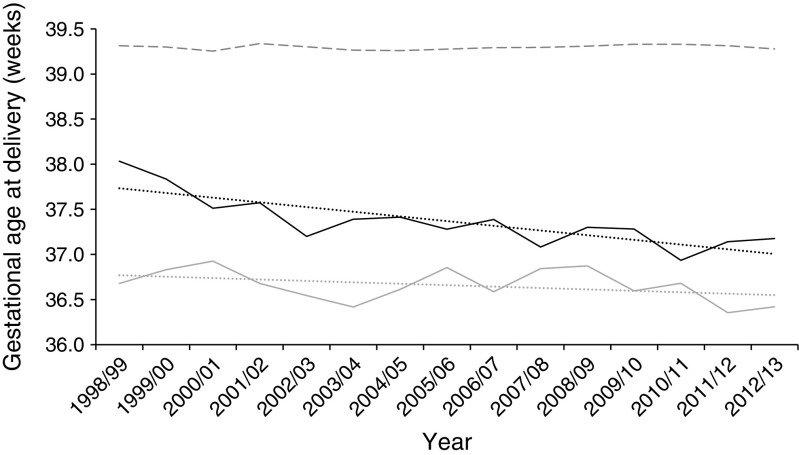
Trends in gestational age at delivery according to diabetes diagnosis. Light grey solid line, type 1 diabetes; black solid line, type 2 diabetes; grey dashed line, population without diabetes; dotted lines in corresponding colours show trends

Delivery occurred at an earlier mean gestation in type 1 and type 2 diabetes, falling slightly from 36.7 weeks in 1998/1999 to 36.4 weeks in 2012/2013 for type 1 diabetes (*p* = 0.03), and from 38.0 to 37.2 weeks in type 2 diabetes (*p* < 0.001) (Fig. 2). The proportion of women delivering preterm increased for both groups, from 34.1% to 42.4% for type 1 diabetes, with a dramatic increase in type 2 diabetes from 11.9% to 25.5% (*p* < 0.005). Very preterm deliveries (under 32 weeks) were uncommon (Table 1) and showed no significant trend. The proportion of singleton deliveries involving Caesarean section increased significantly (*p* < 0.001) for the non-diabetic population from 6.8% to 11.8% for ELCS and from 11.8% to 15.1% for EMCS (ESM Figs 3 and 4). ELCS and EMCS rates were far higher in mothers with type 1 and type 2 diabetes (*p* < 0.001), with the same upward trajectory, despite higher initial rates in women with type 2 diabetes, and for ELCS in women with type 1 diabetes. The only exception was EMCS in women with type 1 diabetes, for which the already very high rates (40%) remained stable (*p* = 0.042).

**Fig. 3:**
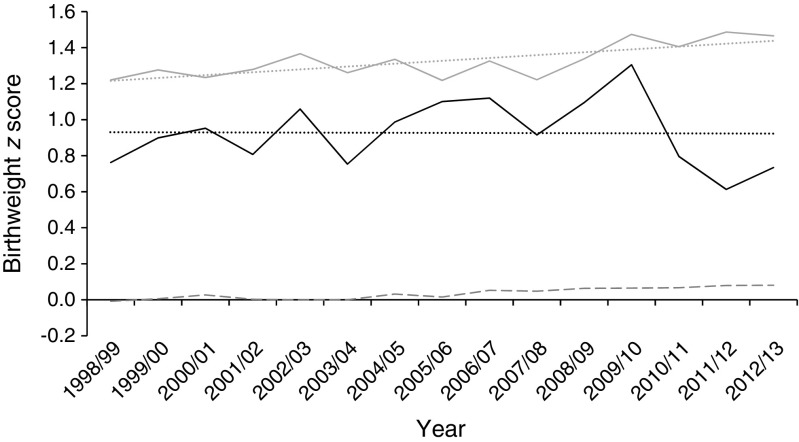
Trends in birthweight *z* score according to diabetes diagnosis. Light grey, type 1 diabetes; black, type 2 diabetes; dashed line, population without diabetes; dotted line in corresponding colours show trends

Adjusted birthweight appeared stable in women with type 2 diabetes, but the *z* score for mean birthweight increased in type 1 diabetes from 1.22 to 1.47 (*p* < 0.001; Fig. 3). This was reflected in the proportion of LGA infants increasing from 47.1% to 56.2% (ESM Fig. 5). LGA rates in type 2 diabetes were stable over time.

Smoking reduced significantly across the time course examined (from 24.1% to 18% in type 1 diabetes, from 22% to 14% in type 2 diabetes, and from 27.9% to 19.3% in the non-diabetic population; *p* < 0.0001). Smoking predicted lower birthweight in type 1 (*p* < 0.001) and type 2 (*p* = 0.003) diabetes, but there was no effect of social deprivation (as represented by the SIMD). The trend to increased *z* score of birthweight in women with type 1 diabetes over time was attenuated by the addition of smoking as an explanatory variable, but it remained significant (*p* < 0.02).

## Discussion

Birthweight, prematurity, operative delivery and perinatal mortality represent key outcome measures in the management of pregnancy complicated by diabetes. It is a concern, then, that stillbirth and perinatal mortality rates appear to be stable over time in our population whereas, at least for type 1 diabetes, birthweight is increasing. Higher rates of operative delivery and falling gestational age at delivery suggest that this is either reflected by, or despite, increasing obstetric intervention.

Our stillbirth rates in type 1 diabetes are intermediate between 2002/2003 data from England and Wales (25.8) [2] and more recent data from 2015 (10.7, for singleton pregnancy only) [14], but are similar to slightly older Swedish data showing 15 stillbirths per 1000 deliveries (1991–2003), and are lower than in other national surveys [10, 22]. Absolute numbers are thankfully small and may influence the ability to detect downward trends. Although our stillbirth rates in type 1 diabetes are not formally significantly different from those for 2015 in England and Wales, they raise concerns that we are not experiencing the dramatic improvement suggested for those countries. There may be greater concern in type 2 diabetes, where the rate is significantly higher than that observed in the recent England and Wales survey (10.7), albeit collected across a much wider range of years. Notably, the difference appears to relate to stillbirth rather than neonatal mortality, which is not changing greatly in England and Wales (for type 1 diabetes 9.6 per 1000 in 2002/2003 and 8.1 in 2015; for type 2 diabetes 9.5 in 2002/2003 and 11.4 in 2015) [2, 14] and is highly comparable to Scottish rates.

Our rates of obstetric intervention are high, significantly so compared with equivalent data from France, Sweden and the Netherlands (44.3–58.9%) [1, 10, 22]. They are also slightly higher than figures for England and Wales, at 66% for type 1 and 56% for type 2 diabetes [14]. The rates of ELCS in type 1 and type 2 diabetes, and most strikingly EMCS in type 2 diabetes, are increasing. Although the proportions of EMCS in type 2 diabetes (29%) we report here appear very similar to those from England and Wales, this compares with a figure of just 10% in the Netherlands [11].

Average birthweight is greatly increased in the offspring of mothers with type 1 and type 2 diabetes—despite the best efforts of individuals and their clinicians. There is an acknowledged, major unmet need to improve glycaemia in pregnancy, particularly in women with type 1 diabetes. The ‘average’ baby born to a mother with type 1 diabetes in Scotland has a birthweight just above the 90th percentile, which is modestly increased compared with 46.4% in England and Wales. Our rates of LGA infants in mothers with type 2 diabetes appear higher (38% vs 24%) [14]. We do not have complete data on important factors such as ethnicity, which will have become more diverse, particularly for type 2 diabetes. However, census data show a greater than 96% white population nationally, suggesting only a moderate influence, at least for type 1 diabetes [23]. Customised centiles were used in England and Wales, which account for maternal height and weight and make direct comparisons difficult.

A particular strength of our data is the ability to look at temporal change. A number of observed trends might have been expected. Although diabetes in pregnancy remains relatively uncommon (1 in 178 births in our data), the prevalence of both type 1 and particularly type 2 diabetes complicating pregnancy is increasing, which may reflect a higher prevalence of obesity, advancing maternal age and modest increases in the size of ethnic at-risk populations [23, 24]. The prevalence of type 1 diabetes overall is also increasing [25]. Although still representing just a small fraction of the overall obstetric population, these increases have important resource implications for service delivery in specialist clinics. The increase in duration of both type 1 and type 2 diabetes is also of importance, as this will probably translate into an increased prevalence of microangiopathy, with an associated risk of placental insufficiency [26].

Other trends may be less expected. Mothers with type 1 diabetes and type 2 diabetes are being delivered earlier, the rate of operative delivery is increasing and, despite this, adjusted birthweight is increasing. In the non-diabetic population, gestation at delivery appears stable, with only small increases in birthweight (<0.1SD across 15 years) but rates of ELCS and EMCS are increasing significantly. We do not have granular data on the clinical reasons for choosing Caesarean section, but this is worthy of further investigation. Similar trends in ELCS rates across groups suggest that general obstetric practice is changing, but this does not explain the rising EMCS rates in type 2 diabetes. It is noticeable that, in England and Wales, EMCS rates in women with diabetes are decreasing (37.6% in 2003/2004, 30% in 2013) [6, 13].

The most recent proportions of LGA infants also appear higher than equivalent figures for England and Wales [14], and others have noted similar increasing trends. In Sweden, the proportion of LGA babies was reported as 23%, 31% and 47% in successive reports of cohorts in their populations of 1982–1985, 1991–2003 and 1998–2007 [22, 27]. Interestingly, this was not attributed to deteriorating glycaemic control, which was probably becoming tighter. We are unable to provide standardised data on glycaemic control over the whole time period but note that we would not expect a temporal deterioration, as this was not observed in the national surveys conducted in 1998/1999 and 2003/2004 [7]. Smoking causes a reduction in birthweight, and happily has reduced significantly in pregnancy in our population over the time course of the study. Accounting for this may provide an explanation for some of the rise in *z* scores in type 1 diabetes. A standard measure of social deprivation did not influence birthweight in women with type 1 or type 2 diabetes. This is of some importance as LGA carries an increased risk of complications in pregnancy [28], although the relative influences of increased absolute size, relative overgrowth and gestational age at delivery warrant further investigation.

The main strengths of our study are its large scale and the fact that it covered all pregnancies in Scotland, thereby avoiding selection bias. We used routinely collected data subject to regular quality assurance checks, and their quality is high. Registration of individuals into SCI-Diabetes occurred in the various Scottish health boards between 2003 and 2006, including an upload of previously held electronic records on current patients in those years. Women who delivered before 2003 and who left Scotland before data entry into SCI-Diabetes may not have been included. Our data collection appears robust, with a slightly higher number of deliveries ascertained in 2003/2004 than from the previous paper audit (172 vs 165) [7], and 96% of deliveries ascertained compared with the 1998/1999 audit [3]. It would appear that the population of women with diabetes is relatively stable, and that ascertainment is successful. The development of similar national audits, most notably in England and Wales, which included 86% of consultant-led obstetric units, is beginning to allow meaningful comparisons of outcomes between countries [14].

As we used pseudonymised data, we cannot directly compare inclusion in the previous surveys case by case. Results from these reported a higher *z* score for this cohort (1.64 in 2003/2004 compared with 1.34 for that year). This simply reflects the standard used: the previous national surveys used data on births from a single centre between 1979–1983, whereas the present study used a contemporaneous, whole-population standard [7]. Finally, we are confined to data that have been electronically captured. This has a major strength in avoiding under-reporting of adverse outcomes by individual units, and the reporting of outcomes such as weight, delivery method and gestational age is known to be robust.

We also acknowledge a weakness of this method in that we are unable to measure some of the measures of care assessed in the previous surveys. Specifically, factors such as attendance at prepregnancy clinics are not recorded, and we lack detailed information on congenital anomalies and neonatal intensive care admissions across the study period. Prenatal glycaemic control has previously been shown to be unchanged between 1998/1999 and 2002/2003 but was suboptimal, with only 54% of women having documented preconceptual HbA_1c_ values, and with average levels 30% above normal [7]. Inadequate preparation for pregnancy may lead to higher rates of congenital malformation and pregnancy loss. It would be of interest to be able to account for other maternal factors such as BMI and gestational weight gain, which may affect infant growth and placental function. These are unlikely to have improved over time [25, 29]. Complications of pregnancy, especially hypertensive disorders, may explain our trends in timing and mode of delivery.

Pregnancy for women with diabetes remains high risk, and much is still to be understood regarding causes and effective interventions for adverse outcomes. As has been shown for other aspects of diabetes care [30], a comparison of rates of complications across different countries and healthcare systems offers an important opportunity to understand the potential for improvement both in Scotland and on a wider international level. This will include concentration on improving prepregnancy care, efforts to improve glycaemic control and best obstetric practice to reduce risk.

## Electronic supplementary material

ESM Figures (PDF 261 kb)

## Data Availability

The datasets used for this study are not publicly available due to confidentiality of clinical systems. The SMR02 is held by ISD Scotland, and requests for access should be directed to ISD Scotland. The SDRN holds SCI-Diabetes records.

## Abbreviations

ELCS: Elective Caesarean section
EMCS: Emergency Caesarean section
ISD: Information Services Division
LGA: Large for gestational age
SCI-Diabetes: Scottish Care Information-Diabetes
SDRN: Scottish Diabetes Research Network
SIMD: Scottish Index of Multiple Deprivation
SMR02: Scottish Morbidity Record 02
