# Supplementary material for: Diabetes and pregnancy: national trends over a 15 year period
Source: Diabetologia. 2018 Jan 11;61(5):1081–8. doi: 10.1007/s00125-017-4529-3 (PMC6448996; doi:10.1007/s00125-017-4529-3)

Electronic supplementary material

Diabetes and pregnancy: National trends over a 15 year period

ESM Fig. 1 Trends in perinatal mortality according to diabetes diagnosis

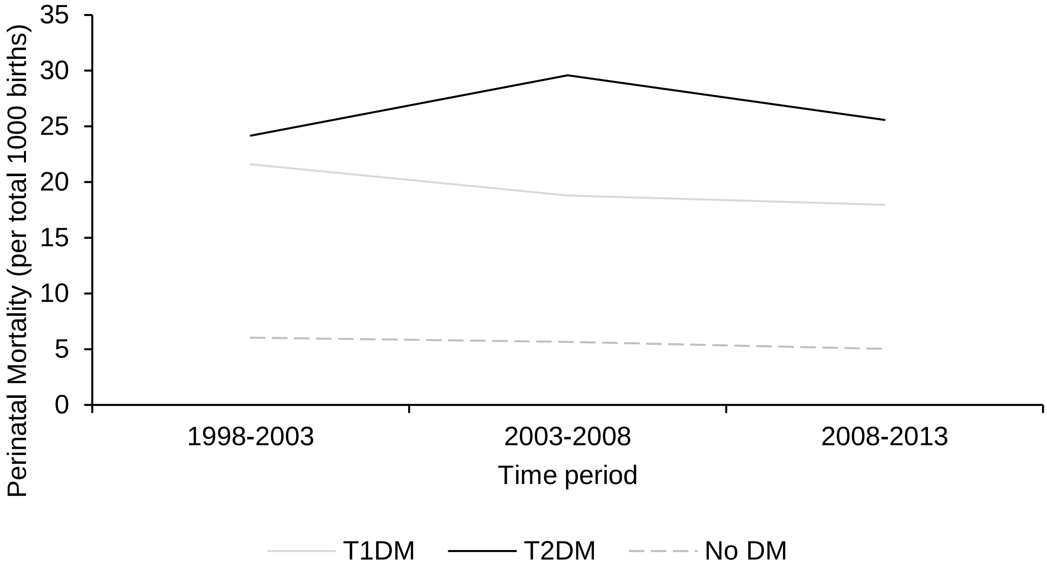

ESM Fig. 2 Trends in maternal age at delivery according to diabetes diagnosis

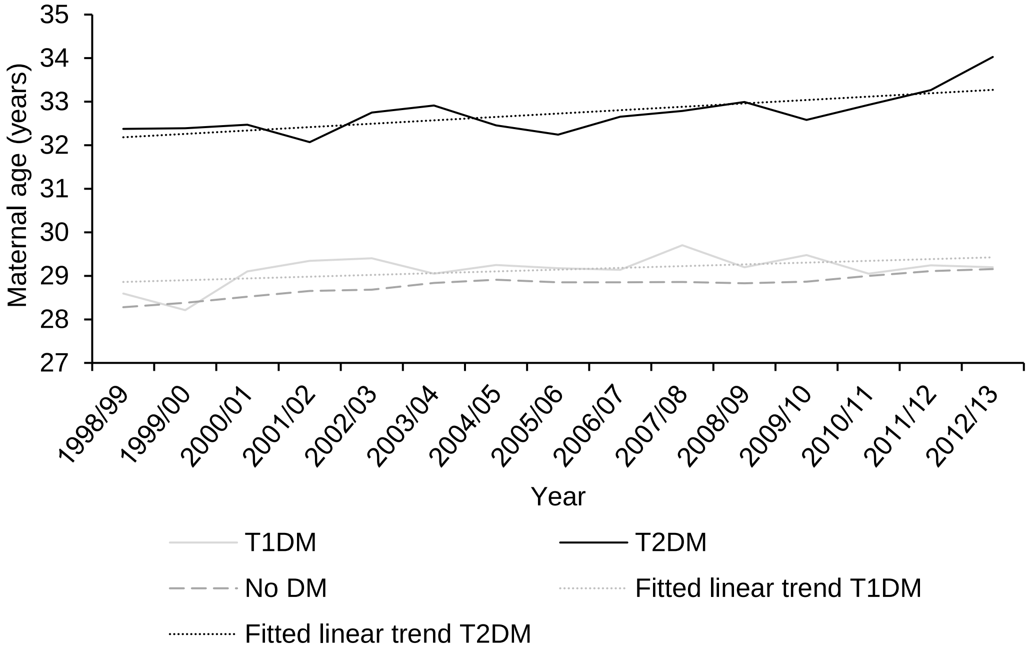

ESM Fig. 3 Trends in elective caesarean section rates according to diabetes diagnosis

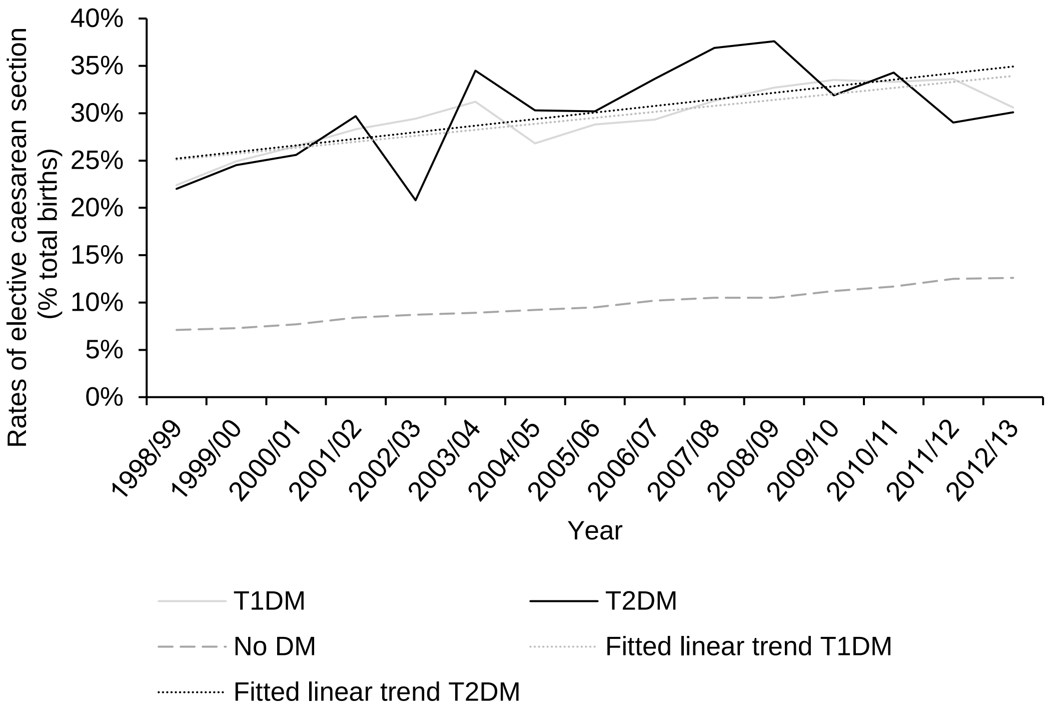

ESM Fig. 4 Trends in emergency caesarean section rates according to diabetes diagnosis

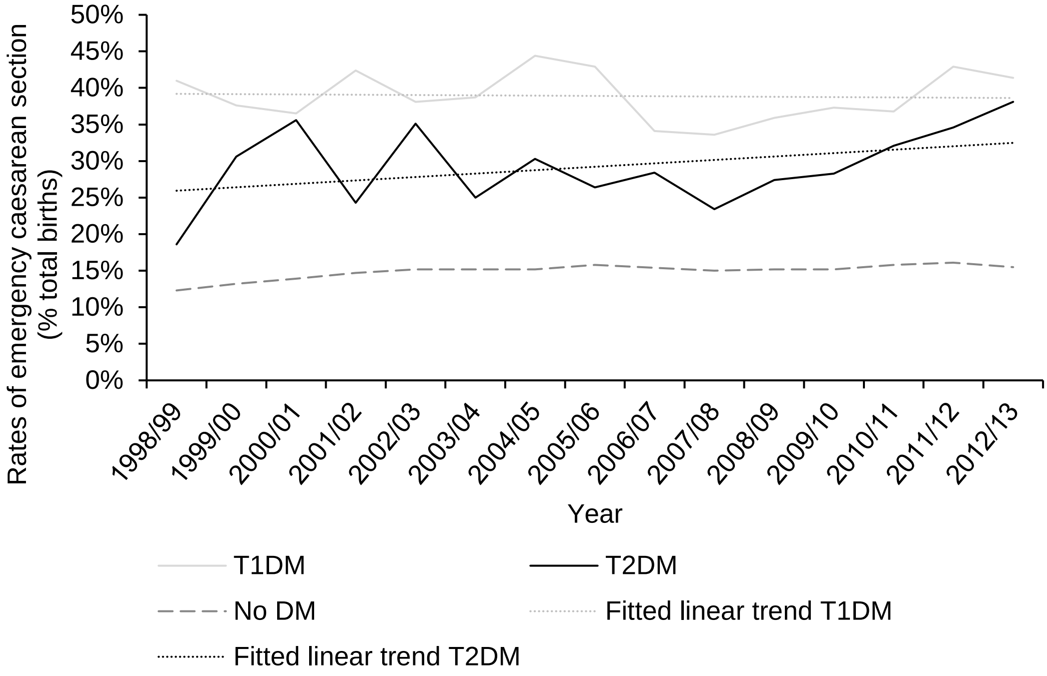

ESM Fig. 5 Trends in rates of LGA infants according to diabetes diagnosis

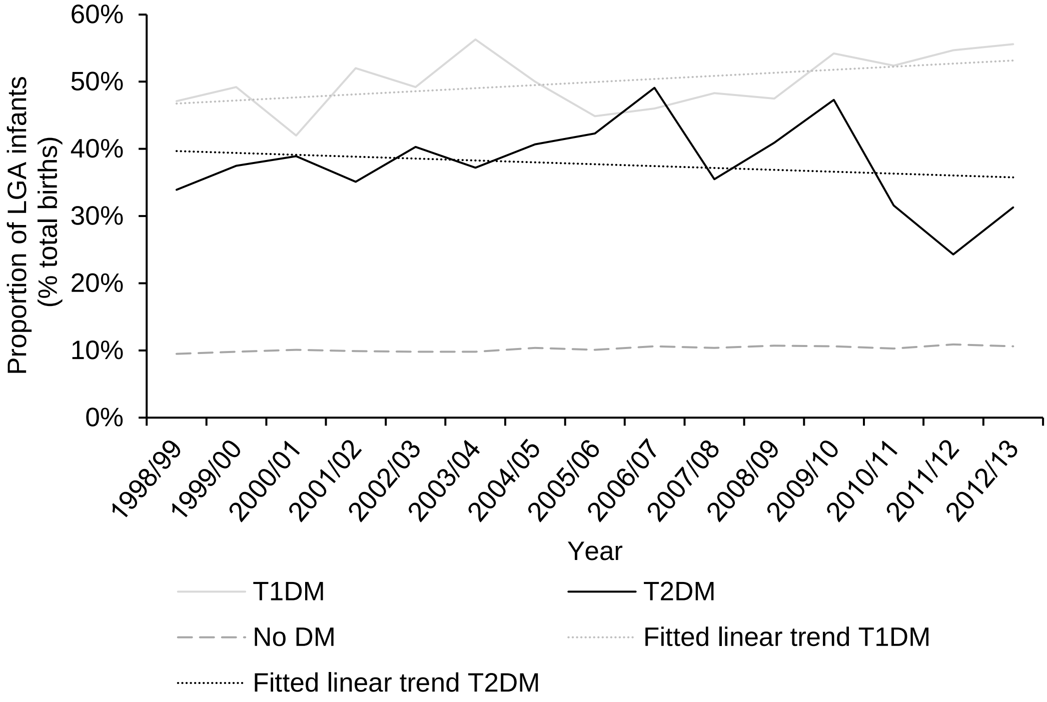

Supplement: Supplementary file 1 — (PDF 261 kb) [file 125_2017_4529_MOESM1_ESM.pdf]
